# Transsacral excision with pre-operative imatinib mesylate treatment and approach for gastrointestinal stromal tumors in the rectum: A report of two cases

**DOI:** 10.3892/ol.2014.2406

**Published:** 2014-08-01

**Authors:** LI-FENG SUN, JIN-JIE HE, SHAO-JUN YU, JING-HONG XU, JIAN-WEI WANG, JUN LI, YONG-MAO SONG, KE-FENG DING, SHU ZHENG

**Affiliations:** 1 Department of Surgical Oncology, The Second Affiliated Hospital, Zhejiang University School of Medicine, Hangzhou, Zhejiang 310009, P.R. China; 2 Department of Pathology, The Second Affiliated Hospital, Zhejiang University School of Medicine, Hangzhou, Zhejiang 310009, P.R. China

**Keywords:** rectal gastrointestinal stromal tumor, imatinib mesylate, transsacral resection, neoadjuvant treatment

## Abstract

Gastrointestinal stromal tumors (GISTs) are rare in the rectum. Radical surgery, such as an abdominoperineal resection, is necessary for large rectal GISTs, which can result in the loss of function of involved organs. Imatinib mesylate can be used as perioperative therapy and may reduce tumor size, and it is now approved for use in the adjuvant therapy of locally resected anorectal GISTs. The present study describes two cases of large rectal GISTs, for which abdominoperineal resections were initially planned. The two patients received pre-operative imatinib mesylate treatment, and the therapeutic response was assessed by magnetic resonance imaging. Finally, transsacral local resection was successfully performed for these two GISTs. A macroscopically complete resection was achieved, and microscopically, the resection margin was negative. One patient experienced the complication of rectal leakage, which was successfully managed by drainage. No recurrence occurred in the two patients after more than two years. Pre-operative imatinib mesylate therapy with subsequent transsacral local resection for selected rectal GISTs is a feasible treatment modality and can prevent extended surgery.

## Introduction

Gastrointestinal stromal tumors (GISTs) are unusual mesenchymal tumors that are commonly found in the stomach (60–70%), and are also found in the small intestine (20–25%); only 5% of all GISTs originate in the rectum. Complete surgical resection is the main therapy for patients with resectable GISTs (1). Patients with rectal GISTs usually undergo extensive procedures, such as abdominoperineal resections (APRs) or low anterior resections (LARs), which may have little benefit in a number of cases, particularly when considering the fact that there is no evidence that extensive surgery prolongs survival or delays recurrence (2,3).

Local excision of anorectal tumors includes the use of transrectal, transsacral and transvaginal approaches (4–6). However, less invasive approaches for the local resection of rectal GISTs are often inadequate due to the size of the mass and its exophytic growth.

In total, 80–95% of GISTs typically express cluster of differentiation (CD)117, a tyrosine kinase growth factor receptor (c-KIT), which can be detected immunohistochemically in order to discriminate GISTs from other mesenchymal gastrointestinal neoplasms (7,8). c-KIT also serves as the target for drug therapy with imatinib mesylate (IM; Glivec^®^), a c-KIT and platelet-derived growth factor receptor (PDGFR)-α inhibitor. IM is now the standard treatment for patients with locally unresectable or metastatic GISTs, and is approved for use in the adjuvant therapy of resectable GISTs. A previous study concluded that pre-operative IM for rectal GISTs is associated with improved surgical margins, and disease-free and overall survival (9).

There are a few studies that have focused on IM adjuvant therapy; these studies have found that following local resection for rectal GISTs, IM is better than, or at least not inferior to, radical surgical LAR or APR (9–11). The present study reports two cases of rectal GISTs that were treated by IM adjuvant therapy and subsequent transsacral local resection. The study was approved by the Ethics Committee of The Second Affiliated Hospital, Zhejiang University School of Medicine (Hangzhou, China), and written informed patient consent was obtained.

## Case report

### Case 1

A 38-year-old male was referred to The Second Affiliated Hospital with the chief complaint of a change in stool shape that had been apparent for two months. The patient’s past medical history was unremarkable. A clinical examination did not detect any palpable abdominal masses. A digital examination of the rectum revealed a mass of ~5 cm in diameter on the anterior rectal wall, ~5 cm above the anal verge. The mass was hard, elastic and immobile, with a smooth, high tension surface. Routine laboratory tests of the serum and urine showed no abnormalities, while the analysis of tumor markers also returned normal results.

**Figure 1:**
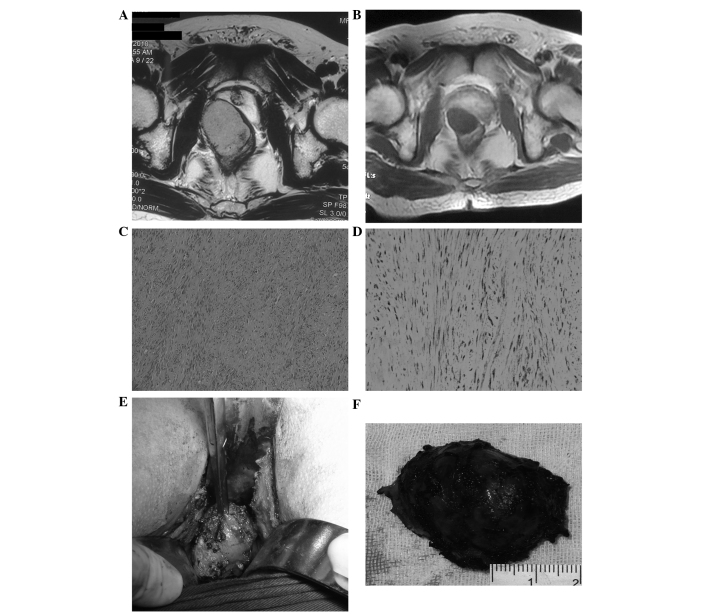
Comparision of pre- and post-IM magnetic resonance imaging (MRI) and tumor tissue in the patient of case 1 who underwent neoadjuvant imatinib mesylate (IM) therapy and transacral resction. (A) MRI prior to IM therapy revealed a 4.9×3.6-cm tumor, with a clear boundary. The tumor exhibited extramural growth on the right anterior wall of the lower rectum, with compression displacement of the prostatic gland. (B) MRI following 7 months of neoadjuvant imatinib therapy demonstrating a 3.3×2.3-cm residual tumor. (C) Biopsy specimen prior to neoadjuvant therapy showing tumor spindle cells (hematoxylin and eosin; magnification, ×100). (D) Immunohistochemical staining for c-kit was positive (c-Kit; magnification, ×200). (E) The transacral intraoperative resection view. The tumor was easily exposed and incised. (F) Gross specimen demonstrating a complete local resection.

Magnetic resonance imaging (MRI) revealed a solitary tumor measuring 4.9×3.6 cm, with a clear boundary. The tumor exhibited extramural growth on the right anterior wall of the lower rectum, with compression displacement of the prostatic gland, but there was no evidence of either pelvic lymphadenopathy or distant metastasis (Fig. 1A). Transrectal ultrasound-guided biopsy samples showed the presence of a spindle cell tumor and strong immunohistochemical positivity for CD117 (Fig. 1C), CD34 and discovered on GIST-1. However, the samples were negative for α-smooth muscle actin (SMA) and desmin. From the results of these examinations, a rectal GIST was diagnosed.

Due to the size and localization of the lesion, IM neoadjuvant therapy was recommended. Therefore, the patient received a single daily dose of 400 mg Glivec for 7 months and was followed up within 3 months by CT or MRI scans to assess the effects. During IM therapy, the tumor continued to shrink (from 4.9×3.6 to 3.3×2.3 cm) (Fig. 1B), with the only side-effect being mild fatigue, and no evidence of progression. Subsequent to 7 months of IM therapy, CT and MRI scans showed no further significant change in tumor size. Therefore, the patient underwent a transsacral local resection (Fig. 1E).

The tumor, which measured 3.5×3 cm, was solid with a clear boundary on the cut sections (Fig. 1F). Histopathological examination revealed that in local areas, the tumor existed with hyaline degeneration of the tumor cells, with <3 mitoses per 50 high-power fields (HPFs). The resection margins were uninvolved on all sides, and there was no lymph node metastasis. Following the surgery, the patient suffered the complication of rectal leakage, which was successfully managed by drainage. To date, no recurrence has been observed in the 24-month follow-up period, subsequent to 5 months of additional post-operative IM treatment.

### Case 2

**Figure 2:**
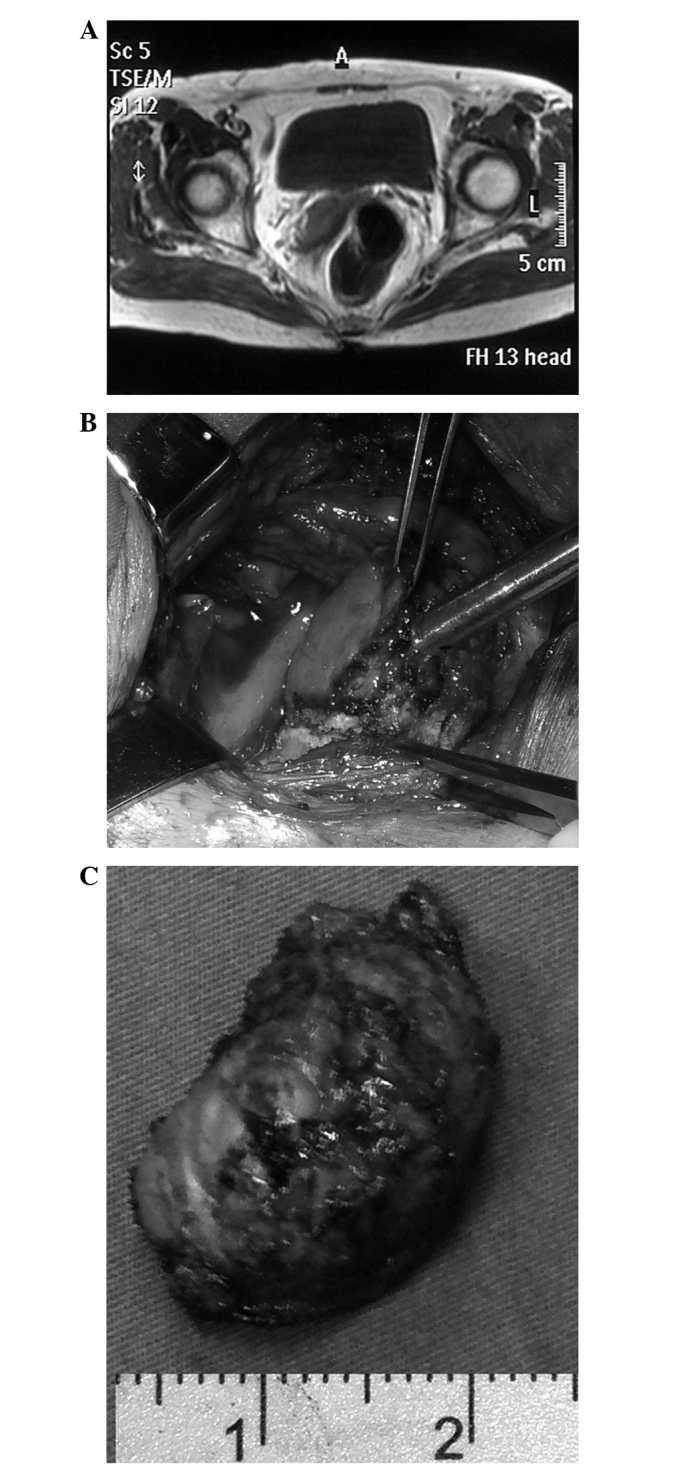
Magnetic resonance imaging (MRI) and tumor tissue in case 2. (A) MRI prior to imatinib mesylate (IM) therapy revealing a solitary tumor measuring 4.5×4.0 cm, with a clear boundary, and exhibiting extramural growth on the right anterior wall of the lower rectum, with compression of the wall of the vagina. (B) The transacral intraoperative resection view. The tumor was easily excised. (C) Gross specimen demonstrating a complete local resection.

A 76-year-old female presented with a 3-month history of a change in stool shape. A pelvic MRI scan showed a solid tumor measuring 4.5×4.0 cm, with clear boundary. The tumor exhibited extramural growth on the right anterior wall of the lower rectum, with compression of the wall of the vagina (Fig. 2A).

A transrectal ultrasound-guided biopsy showed a spindle cell stromal tumor with >5 mitoses per 50 HPFs, and immunohistochemical positivity for CD117 and CD34. The neoplastic cells were negative for α-SMA and desmin. The pathologic findings were consistent with a high-risk GIST

The patient began therapy with 400 mg IM once daily, and was followed-up within 3 months with transrectal ultrasound and MRI scans. Subsequent to IM therapy for 3 months, the tumor had shrunk to 3.0×2.0 cm in size and there were no side-effects or evidence of progression. The patient requested surgery to remove the tumor, and therefore underwent a transsacral local resection (Fig. 2B).

The tumor, which measured 2.3×1.9 cm, was soft and had a light-yellow parenchyma, with focal cysts on the cut sections (Fig. 2C). Histopathological examination revealed that there was local necrosis of the tumor cells. The tumor cells were strongly positive for CD117 and CD34, and negative for SMA and S-100 protein. There were <3 mitoses per 50 HPFs. To date, the post-operative course has been satisfactory, and there has been no recurrence for 28-months without IM treatment.

## Discussion

GISTs are the most common mesenchymal tumor of the gastrointestinal tract, and are likely to arise from the precursor interstitial cells of Cajal. GISTs are common in the stomach (60–70% of cases) and small intestine (30%), and occur rarely in the rectum (5%), esophagus, colon, pancreas, appendix, omentum, mesentery and retroperitoneum (1,2).

The symptoms of a rectal GIST do not generally differ from those of other rectal tumors. Occasionally, no symptoms are present. As for diagnosis of rectal GIST, digital examination of the rectum, transanal ultrasound and colonoscopy are essential and part of the same workup that is used for other rectal masses. Pre-operative biopsies are a vital part of the diagnosis of a GIST, as they provide immunohistochemical data, such as the positivity for CD117 and CD34, and the mitotic count. In total, ~95% of GISTs express CD117, and ~70% are CD34-positive (12). Furthermore, MRI or CT scans are required to determine the extent of local invasion and to detect the possible metastases. The size, site and mitotic index of the GISTs are the most used prognostic factors and aid risk stratification of recurrence. The National Institute of Health has defined those lesions with a diameter of >10 cm, a mitotic rate of >10/50 HPF or a diameter of 5 cm, and a mitotic rate of >5/50 HPF as the tumors that are at a high-risk of metastasis (13).

Surgery is the primary treatment of choice for patients with localized or potentially resectable GISTs. Various surgical procedures may be considered, including local excision, LAR and APR with total mesorectal excision (TME). The only potentially curative treatment for GISTs is complete surgical resection with negative tumor margins (1,12). Compared with rectal adenocarcinoma, rectal GISTs exhibit two specific features that may significantly affect surgical management. Firstly, metastases are extremely rare in the locoregional lymph nodes, and secondly, GISTs typically show a tendency to grow away from the intestinal lumen (2). So for the surgical management of a large GIST arising in the lower rectum, radical surgery, including LAR and APR with TME, may have little benefit (15). A previous study found that GISTs >5 cm in diameter that were removed by APR, LAR or local excision demonstrated no significant differences with regard to survival. The study suggested that the natural history of these GISTs partly cancels out the benefit of radical surgery (2).

The surgery most frequently proposed for the local excision of anorectal tumors is a transrectal approach with application of various dilators. This approach is most suitable for tumors whose distal margin from the dentate line is ~3 cm (6,16). Other possible approaches for local excision include the transvaginal route, and the transcoccygeal or trans-sphincteric approach. Transvaginal local excision for rectal carcinoma has also been performed in patients with T1 and T2 rectal cancers. The average distance from the dentate line that best fits this approach is ~4 cm. The possible complication of a rectovaginal fistula occurs at a low rate and is treated conservatively (16).

Transcoccygeal (transsacral) excision, is suitable for higher lesions (average distance from the dentate line, 5 cm) located in the posterior wall of the rectum (5,17,18). This location requires a paracoccygeal incision between the anus and coccyx, an S5 or coccygeal transection, and an incision of Waldeyer’s fascia, with exposure of the perirectal fat. The tumor may be excised through a wedge resection or even a segmental resection with an end-to-end anastomosis. However, certain post-operative complications have also been described, including wound infections, urinary retention, fecal fistulae, fecal incontinence and hemorrhage (5,19).

In addition, the trans-sphincteric approach is well suited for exophytic GISTs located anteriorly and in the lower third of the rectum. This approach requires the sphincter to be divided; the exposure of the lower rectum is similar to a transcoccygeal approach, but there is rising concern regarding long-term continence problems (4,16).

However, less invasive approaches for local resection of rectal GISTs are often inadequate due to the size of the mass and its exophytic growth. The larger the tumor, the more difficult it is to obtain tumor-free margins. An alternative approach would be the use of pre-operative IM therapy for large rectal GISTs, which may result in tumor shrinkage (20). In a previous study, neoadjuvant therapy with IM was used prior to local excision via the Kraske approach (17), which showed that preoperative IM therapy resulted in the shrinkage of GISTs and exhibited a clear benefit with regard to local excision.

In total, 80–95% of GISTs typically express CD117, a c-kit proto-oncogene, which can be detected immunohistochemically in order to discriminate GISTs from other mesenchymal gastrointestinal neoplasms (7,8). c-kit also serves as the target for drug therapy with IM, a c-kit and PDGFR-α inhibitor. IM is now standard treatment for patients with locally unresectable or metastatic GISTs, and is approved for use in the adjuvant therapy of resectable GISTs.

The 10 European Organization for Research and Treatment of Cancer Soft Tissue and Bone Sarcoma Group sarcoma centers (19) and the Radiation Therapy Oncology Group (RTOG) phase II study (RTOG0132) (22) evaluated and analyzed the safety and efficacy of neoadjuvant IM for patients with locally advanced primary GISTs. The results showed indicated excellent long-term results in locally advanced GISTs treated with neoadjuvant IM in routine practice and found that the complications of surgery and IM toxicity were minimal. The approach is therefore feasible

The recommended duration of pre-operative IM therapy in the adjuvant setting is not known. The median time to the best response in all responding patients was ~4 months (107 days), and the majority of responses occurred within 9 months of treatment (23). Verweij *et al* (23) recommend that studies on neoadjuvant IM therapy should be designed with the duration of treatment ranging between 4 and 6 months. In the present cases, surgery was performed following 3 to 7 months of treatment, in order for the tumor shrinkage to have stabilized.

Several case studies have demonstrated that the use of pre-operative IM enables organ-sparing surgery and improves surgical outcomes in patients with rectal GISTs (17,24,29).

There are a few studies that have shown that the use of IM adjuvant therapy and subsequent local resection is better than, or at least not inferior to, LAR or APR for anorectal GISTs (9,10,11). The present study reports the cases of two rectal GISTs that were treated by IM adjuvant therapy and subsequent transsacral local resections. There were no severe complications, except a slight fistula, and no recurrence and metastasis occurred after >2 years of follow-up. Neoadjuvant IM therapy following the local resection of anorectal GISTs in the literature is also summarized in the present study, and the results are shown in Table I.

The literature review found that radical surgery did not always generate a better outcome than local excision for anorectal GISTs. In a study of 144 cases of anorectal GISTs, Miettinen *et al* (2) found that there was no significant difference in the survival rates between patients who underwent radical surgery and local excision. Radical surgery, including LAR or APR, possibly affected or sacrificed anal sphincter function and was associated with high mortality and morbidity. The natural history of these tumors may partly cancel out the benefit of radical surgery.

Jakob *et al* (9) concluded that if pre-operative IM was used, it was associated with improved surgical margins and local disease-free, total disease-free and overall survival. Local excision did not incur elevated local recurrence rates. The study found that 5 out of 21 local excisions for anorectal GISTs incurred local recurrence, as these patients underwent local excision with positive margins. Complete resection is recommended to achieve local disease control. The study also found that 5 out of 39 patients without IM therapy incurred metastasis (9).

Laparoscopic surgery has been a breakthrough in the field of rectal cancer surgery. Fujimoto *et al* (25) reported the cases of five patients who were treated by a combination of neoadjuvant IM therapy and laparoscopic sphincter-preserving surgery [intersphincteric resection (ISR) or modified ISR] for a large rectal GIST. All patients underwent complete surgical resection macroscopically and microscopically, including one case with a complete response, thereby avoiding a radical excision and preserving the anus (25).

From the present study and the literature, it can be observed that IM therapy plus local excision does not incur severe complications and colorectal dysfunction. Therefore, the pre-operative use of IM combined with subsequent local resection is a viable therapeutic option for anorectal GISTs and allows less extensive resections. In the present study, there were no severe complications, except a slight fistula, and no recurrence and metastasis occurred after more than two years of follow-up. The key point of this therapy strategy is to obtain a tumor-free margin and to preserve the function of the anal sphincter.

## Figures and Tables

**Table I:** Summary of the anorectal gastrointestinal stromal tumors cases from the literature that underwent neoadjuvant IM therapy following local resection.

First author, year (ref.)	Cases, n	Local excision	Pre-operative IM, n	Post-operative IM, n	Risk of recurrence	Outcome
Fujimoto *et al*, 2013 (25)	5	Laprascopic ISR	5	3	High for 3	ANED
Agaimy *et al*, 2013 (10)	16	6 cases	3	7	High for 13	Incomplete resection associated with high local recurrence rates
Centonze *et al*, 2013 (4)	2	2 cases	2	2	High	ANED
Tielen *et al*, 2013 (11)	32	8 cases	22	Yes	N/A	Pre-operative IM did not lead to less extensive surgery
Jacob *et al*, 2012 (9)	39	21 cases for local excision	16	N/A	N/A	5 recurrence, 5 metastasis cases
Lagos *et al*, 2012 (26)	1	Transanal	No	Yes	High	ANED
Wang *et al*, 2011 (17)	3	Transsacral	Yes	N/A	N/A	ANED
Hara *et al*, 2011 (27)	1	Transvaginal	No	No	High	ANED
Matsushima and Kayo, 2007 (18)	2	Transsacral	N/A	N/A	Medium	ANED
Gervaz *et al*, 2008 (28)	1	Transsacral	No	No	High	N/A
Shelly *et al*, 2005 (29)	1	Transanal	Yes	N/A	High	ANED
Miettinen *et al*, 2001 (2)	144	24 cases	No	No	N/A	No difference in survival between radical and local resection
Present study	2	Transsacral	2	2	High	ANED

IM, imatinib mesylate; ANED, alive with no evidence of disease; ISR, intersphincteric resection; N/A, not applicable.
